# Validation of a short form Wisconsin Upper Respiratory Symptom Survey (WURSS-21)

**DOI:** 10.1186/1477-7525-7-76

**Published:** 2009-08-12

**Authors:** Bruce Barrett, Roger L Brown, Marlon P Mundt, Gay R Thomas, Shari K Barlow, Alex D Highstrom, Mozhdeh Bahrainian

**Affiliations:** 1 Department of Family Medicine, University of Wisconsin-Madison 1100 Delaplaine Ct., Madison, WI 53715 USA; 2 School of Nursing, University of Wisconsin-Madison K6/287 Clinical Science Center, Madison, WI 53792 USA

## Abstract

**Background:**

The Wisconsin Upper Respiratory Symptom Survey (WURSS) is an illness-specific health-related quality-of-life questionnaire outcomes instrument.

**Objectives:**

Research questions were: 1) How well does the WURSS-21 assess the symptoms and functional impairments associated with common cold? 2) How well can this instrument measure change over time (responsiveness)? 3) What is the minimal important difference (MID) that can be detected by the WURSS-21? 4) What are the descriptive statistics for area under the time severity curve (AUC)? 5) What sample sizes would trials require to detect MID or AUC criteria? 6) What does factor analysis tell us about the underlying dimensional structure of the common cold? 7) How reliable are items, domains, and summary scores represented in WURSS? 8) For each of these considerations, how well does the WURSS-21 compare to the WURSS-44, Jackson, and SF-8?

**Study Design and Setting:**

People with Jackson-defined colds were recruited from the community in and around Madison, Wisconsin. Participants were enrolled within 48 hours of first cold symptom and monitored for up to 14 days of illness. Half the sample filled out the WURSS-21 in the morning and the WURSS-44 in the evening, with the other half reversing the daily order. External comparators were the SF-8, a 24-hour recall general health measure yielding separate physical and mental health scores, and the eight-item Jackson cold index, which assesses symptoms, but not functional impairment or quality of life.

**Results:**

In all, 230 participants were monitored for 2,457 person-days. Participants were aged 14 to 83 years (mean 34.1, SD 13.6), majority female (66.5%), mostly white (86.0%), and represented substantive education and income diversity. WURSS-21 items demonstrated similar performance when embedded within the WURSS-44 or in the stand-alone WURSS-21. Minimal important difference (MID) and Guyatt's responsiveness index were 10.3, 0.71 for the WURSS-21 and 18.5, 0.75 for the WURSS-44. Factorial analysis suggested an eight dimension structure for the WURSS-44 and a three dimension structure for the WURSS-21, with composite reliability coefficients ranging from 0.87 to 0.97, and Cronbach's alpha ranging from 0.76 to 0.96. Both WURSS versions correlated significantly with the Jackson scale (W-21 R = 0.85; W-44 R = 0.88), with the SF-8 physical health (W-21 R = -0.79; W-44 R = -0.80) and SF-8 mental health (W-21 R = -0.55; W-44 R = -0.60).

**Conclusion:**

The WURSS-44 and WURSS-21 perform well as illness-specific quality-of-life evaluative outcome instruments. Construct validity is supported by the data presented here. While the WURSS-44 covers more symptoms, the WURSS-21 exhibits similar performance in terms of reliability, responsiveness, importance-to-patients, and convergence with other measures.

## Background

The common cold is a clinical syndrome resulting from viral infection of the upper respiratory tract. Etiologic agents include rhinovirus, coronavirus, parainfluenza, influenza, respiratory syncytial virus, adenovirus, enterovirus, and metapneumovirus [1-3]. Upper respiratory infection (URI) is extremely common, accounting for up to half of all acute illness episodes[4]. Approximately 70% of the population experiences a cold in a given year, with the age specific incidence approximating 4 to 6 colds per year in children and 1 to 3 per year among adults [5-7]. Incidence rates of viral respiratory infection are higher than clinical colds, as many infections are asymptomatic. The annual economic impact of non-influenza URI is estimated at $40 billion, with more than 40 million days of work and school lost[8].

There are no perfect tools for assessing common cold. Laboratory measures of URI include identification of virus, quantitative viral titer, mucus weight, counts of neutrophils or other white blood cells, and quantitative assay of various cytokines [9-15]. As indicators of immune and inflammatory processes these biomarkers are useful, but none correlate well with illness domains (specific symptoms, functional impairments),[16] and none have been shown to predict important outcomes. The Jackson scale [17-19] (technically an index and not a scale[20]) is the most commonly used questionnaire used for defining and evaluating colds and flu. Jackson's index includes eight symptoms which are rated as absent, mild, moderate or severe by either self-assessment or with clinician/researcher assistance. Jackson's method has been compared to laboratory measures, but has not been psychometrically assessed, and does not include quality of life (QoL) measures. Aside from Jackson, there are no recognized questionnaire instruments able to assess URI illness severity in adults. The CARIFs scale includes QoL items,[21,22] but is designed to assess colds only among children.

The Wisconsin Upper Respiratory Symptom Survey (WURSS) was developed using individual interviews and focus groups among community-recruited people with Jackson-defined colds[23]. Semi-structured interviews included open-ended questions aimed at eliciting terminology and assessing health values related to experienced cold illness. Of more than 150 terms used to define symptomatic or functional impairment, 42 were chosen for inclusion in the WURSS-44[23]. In addition to the 42 specific items, one introductory question assesses global severity, and another final question assesses improvement or deterioration (change-since-yesterday). More information on the WURSS can be found at: .

The first stage of WURSS validation was based on data gathered during monitoring of 150 adults during 1,681 person-days of illness[24]. Factor analysis tentatively identified ten domains. Items assessing activity, quality of life, and functional impairment were rated as equally or more important than items assessing symptom severity. Minimal important difference and responsiveness were assessed following methods of Guyatt et al [25-29]. Using responsiveness and importance-to-patients as guides, we selected best items for inclusion in a short-form, the WURSS-21[24]. Table 1 shows the items in the WURSS-44 and WURSS-21, along with the domains identified previously[24].

**Table 1:** Content of the Wisconsin Upper Respiratory Symptom Survey (WURSS-44)

**Symptoms**	**Symptoms**	**Symptoms**	**Functional impairments**
***1. How sick do you feel today? [Gt]***	12. Body aches **[A]**	23. Swollen glands **[A]**	***34. Think clearly *[F]**

***2. Cough *[C]**	13. Feeling "run down" **[Ti]**	24. Plugged ears **[E]**	35. Speak clearly **[F]**

3. Coughing stuff up **[C]**	14. Sweats **[Sw]**	25. Ear discomfort **[E]**	***36. Sleep well *[F]**

4. Cough interfering with sleep **[C]**	15. Chills **[Sw]**	26. Watery eyes **[O]**	***37. Breathe easily *[F]**

***5. Sore throat *[Th]**	16. Feeling feverish **[Sw]**	27. Eye discomfort **[O]**	***38. Walk, climb stairs, exercise *[F]**

***6. Scratchy throat *[Th]**	17. Feeling dizzy **[O]**	***28. Head congestion *[O]**	***39. Accomplish daily activities *[F]**

***7. Hoarseness *[Th]**	***18. Feeling tired *[Ti]**	***29. Chest congestion *[Ch]**	***40. Work outside the home *[F]**

***8. Runny nose *[N]**	19. Irritability **[O]**	30. Chest tightness **[Ch]**	***41. Work inside the home *[F]**

***9. Plugged nose *[N]**	20. Sinus pain **[Si]**	31. Heaviness in chest **[Ch]**	***42. Interact with others *[F]**

***10. Sneezing *[N]**	21. Sinus pressure **[Si]**	32. Lack of energy **[Ti]**	***43. Live your personal life *[F]**

11. Headache **[Si]**	22. Sinus drainage **[Si]**	33. Loss of appetite **[O]**	***44. Compared to yesterday [Gy]***

*Items selected for WURSS-21 are in bold italics*

Directions for items (2 – 33): "Please rate the average severity of your cold symptoms over the last 24 hours by marking the appropriate circle for each of the following symptoms."

Response options range 0 to 7, with 0 = Do not have, 1 = Very mild, 3 = Mild, 5 = Moderate, 7 = Severe

Directions for items (34 – 43): "Over the last 24 hours, how much has your cold interfered with your ability to..."

Response options are 0 to 7, with 0 = Not at all, 1 = Very mildly, 3 = Mildly, 5 = Moderately, 7 = Severely

Factor analysis for original validation study identified 10 domains: C = Cough; Th = Throat; N = Nasal; A = Aches; Ti = Tired; Si = Sinus/headache; Sw = Sweats/chills/fever; E = Ears; Ch = Chest; F = Functional/activity

Gt = Global severity today; Gy = Global change since yesterday; O = Did not fit within any domain

Our conceptual framework regarding common cold is influenced by works of Jackson, [17-19] Gwaltney, [30-32] Monto,[1,7,33] Eccles,[34,35] and Turner, [36-38] whose works collectively define common cold as a clinical illness syndrome characterized by symptomatic expression caused by viral infection of the upper respiratory tract. We follow the theory of health measurement and instrument validation described by McDowell and Newell[20] and others [39-41]. Our work is influenced by Guyatt et al., [25-28], especially in regard to minimal important difference and responsiveness. WURSS was designed to be an evaluative outcomes instrument, aimed at measuring change over time in patient-valued illness domains. Its greatest value will likely be as a patient reported outcome (PRO) instrument for use in clinical trials.

## Methods

The current study was conceived as a second sample for WURSS validation, and as a chance to compare the WURSS-21 to the WURSS-44. Methods were designed to answer the following questions: 1) How well does the WURSS-21 assess the symptoms and functional impairments associated with common cold? 2) How well can this instrument measure change over time (responsiveness)? 3) What is the minimal important difference (MID) that can be detected by the WURSS-21? 4) What are the descriptive statistics for the area under the time severity curve (AUC), as measured by the WURSS-21? 5) What sample sizes would randomized trials require to detect either day-to-day MID or pre-specified proportional reductions in AUC? 6) What does factor analysis tell us about the underlying dimensional structure of the common cold, as measured by WURSS? 7) How reliable are items, domains, and summary scores represented in WURSS? 8) For each of these considerations, how well does the WURSS-21 compare to the WURSS-44, Jackson, and SF-8?

Our basic methodology was to recruit people early in the course of their colds, then follow them with twice daily self-assessments until their colds resolved, to a maximum of 14 days. Prospective participants responding to advertising or word of mouth were screened on the telephone, then met for informed consent and study enrollment. Half the sample filled out the WURSS-21 in the morning and the WURSS-44 in the evening; the other half completed the questionnaires in reverse order. In addition to the WURSS-21 and WURSS-44, participants filled out the Jackson scale [17-19] every day, and the SF-8 (24 hour recall) daily starting the day after enrollment. The SF-8 is a short form 24 hour recall version of the widely used SF-36, and yields separate summary scores for physical and mental health, calculated using algorithms recommended by the authors[42].

The protocol was approved by the University of Wisconsin Institutional Review Board's Human Subject Committee. Participants were recruited from the community in and around Madison, Wisconsin, using newspaper advertisements, flyers, posters, email messages, a promotional website, and targeted mailings of post cards and letters. Responders to advertisement were screened for eligibility criteria during a pre-enrollment phone interview. Presence and timing of symptom onset was assessed during phone screening and again in person just prior to enrollment. Inclusion required a Jackson score of 2 or higher, with symptom severity rated as 0 = absent, 1 = mild, 2 = moderate, or 3 = severe for each of the eight Jackson symptoms: sneezing, nasal discharge, nasal obstruction, sore throat, cough, headache, malaise, and chilliness. At least one of the first four "cold-specific" Jackson symptoms was required, and none these could have been present for more than 48 hours. Exclusion for allergy was based on a history of allergy combined with current eye or nose itching or sneezing. Exclusion for asthma was based on a history of asthma with current cough, wheezing or shortness of breath. Additionally, people were excluded if either the prospective participant or the enroller felt that any current symptoms were likely due to allergy, asthma, or other non-URI cause.

We defined cold illness to begin with first cold-specific Jackson symptom (nasal or throat), and to continue until the participant reported being "not sick" for two days in a row. Our protocol required that enrollment occurred within 48 hours of the first cold symptom. Participants were required to answer "Yes" to "Do you think you have a cold?" at the enrollment interview. In the morning and evening of each subsequent day, participants answered "How sick do you feel today?" by marking a 0 to 7 Likert-type severity scale, where 0 = Not sick, 1 = Very mildly, 3 = Mildly, 5 = Moderately, and 7 = Severely. Even numbers did not have descriptors. Colds were defined as ending when a participant marked "0 = Not sick" twice in a row on two subsequent days. If this did not occur by the 14^th ^day, participation was terminated. Protocol adherence was supported by regular telephone contact. Questionnaire instruments were returned at an in-person exit interview after the cold ended.

To assess importance-to-patients, we attached the question "How important is this to you?" to each of the WURSS-44 items at enrollment. Participants were told: "Some people may rate one symptom as fairly severe, but not think it is very important, while other, milder symptoms may really bother them. When answering the question, "How important is this to you?" please think about how bothersome a symptom is, or how much you dislike having it." The 5-point response option scale had the descriptors "Not," "Somewhat," and "Very" aligned with the numbers 1, 3 and 5.

Following MID methods attributable to Guyatt et al., [25-29] participants were first asked whether they were "better," "the same," or "worse," compared to the last time they answered the questionnaire. Those considering themselves "better" then rate improvement as: 1) Almost the same, hardly any better at all, 2) A little better, 3) Somewhat better, 4) Moderately better, 5) A good deal better, 6) A great deal better, or 7) A very great deal better. Those saying they were "worse" rate the degree of deterioration on a corresponding 7 point scale.

Operationally, MID is taken to be the average amount of instrument-assessed change for all subjects who rate themselves as "a little better" or "somewhat better"[27,28,43,44]. Guyatt's index of responsiveness is then calculated by dividing this MID by the square root of twice the mean square error (MSE) of stable participants (people who rate interval change as "the same.") Thus, Guyatt's Responsiveness Index is defined as MID/. We have previously adapted these methods for use in common cold,[16,24,45] and have proposed additional strategies for assessing patient-valued outcomes [46-49]. Cohen's standardized effect size and the standard error of measurement (SEM) represent alternative strategies that can be employed to compare change over time.

For acute illness, which has a beginning and an end, area under the curve (AUC) may be an appropriate parameter to consider for the primary outcome for clinical trials. While various strategies such as a fitting of curves or trapezoidal approximation could be used to assess AUC, the current study simply adds daily WURSS scores across all days of documented illness to arrive at the AUC measure reported here.

Factor analysis of the first WURSS validity data set tentatively suggested a factorial structure of ten dimensions[24]. The current study was designed to re-assess the dimensional structure of the WURSS-44, and to explore the structure of the WURSS-21. For both the previous and current studies, the general approach followed methods described by Kroonenberg and Lewis[50]. This approach combines exploratory and confirmatory procedures, using weighted least square estimates employing diagonal weight matrix techniques to seek common factors within empirically derived domains. For the current study, we did not assume that the factorial structure identified in the first WURSS validation effort was inherently sound, but instead started without any *a priori *grouping of items. Realizing that factors and dimensions are rarely orthogonal (truly independent), we allowed for the possibility of factors falling within multiple dimensions. Once best fit dimensional structures were found, construct reliability was estimated using methods originally proposed by Joreskog,[51] developed further by Bollen[52]. All factor analyses were conducted using Mplus Version 5.1[53].

Data were hand entered twice, with resolution of discrepancies by comparison to paper questionnaires. Missing data, disallowed values, and outliers were also hand-checked, and corrected if appropriate. Overall, >98% of intended data was collected. Formal missingness analysis was done for each instrument separately, following the approach set forth by Potthoff[54]. Assumptions were met for missing at random (MAR+),[54] therefore imputation using multivariate techniques was deemed acceptable. Reliability coefficients were calculated using methods of Joreskog[51] and Bollen,[52] with significance tested following Wald[55,56].

To assess item/dimension structure with factor analysis, we chose an iterative combined exploratory and confirmatory strategy, as described by Kroonenberg and Lewis[50].

## Results

The first participant was enrolled on August 11, 2003. The last exited on August 21, 2007. This study was done in parallel with a randomized controlled trial testing echinacea, placebo effects, and doctor patient interaction in common cold[57]. Joint recruitment methods targeted community members with new onset common cold. Of 2,169 responding callers, 534 were enrolled in that trial, and 239 were consented and enrolled in the validation study reported here. Of those enrolled, 230 were monitored through the duration of their colds, for a total of 2,457 person-days covered by this study.

Reasons for exclusion included symptom duration greater than 48 hours (462), allergy or asthma symptoms (50), failure to meet Jackson cold criteria (44), intended use of symptom-modifying medications (33), and subject judged to be unreliable (24). Reasons for non-enrollment of eligible callers included: participant burden (74), failure to return phone calls (65), failure to show up for enrollment (21), "not interested" (17), transportation problems (14), and insufficient compensation (5). Of the nine lost to follow-up, three people never returned phone calls, three reported losing their folders and never came in for their exit, two called to withdraw and never came in for their exit interview, and one person staying at a homeless shelter could not be contacted. Table 2 portrays enrollment, monitoring and sociodemographic characteristics for the population sampled.

**Table 2:** Participant characteristics

**Participants**			Number (per cent)
		Ethnicity*	

**Enrollment**		White	198 (86.0)

Number of calls	2,169	Black	16 (7.0)

Enrolled in other study	534	Hispanic	1 (0.4)

Enrolled in this study	239	Asian	4 (1.7)

Completing protocol	230	Native American	2 (0.8)

		Other/No response	10 (4.4)

**Age and Sex**		**Income**	

Age range	14 to 83	<15 K/yr	91 (39.6)

Mean (SD)	34.1 (13.6)	15 to <25 K/yr	24 (10.4)

	Number, per cent	25 to <50 K/yr	35 (15.2)

Women	153 (66.5)	50 to <75 K/yr	39 (17.0)

Men	77 (33.5)	75 to <100 K/yr	26 (11.3)

		>100 K/yr	11 (4.8)

**Education (highest achieved)**		No response	4 (1.7)

Some HS	9 (3.9%)	**Tobacco use**	

HS degree/GED	54 (23.5%)	Current > 5 cigarettes/day	18 (7.8)

Some college	61 (26.5%)	Current ≤ 5 cigarettes/day	19 (8.3)

College degree	104 (45.2%)	Past	50 (21.7)

		Non-smoker	141 (61.3)

		No response	2 (0.9)

*One person self-identified as both white and Native America

Time from first symptom to enrollment averaged 33.1 hours (SD = 13.4), inter-quartile range (25 to 45). Adding pre-enrollment illness hours to duration monitored (mean = 193.8, SD = 86.9) yields our estimate of mean total illness duration 226.9 hours (SD = 87.5), or 9.45 days. This may be an underestimate of actual average illness duration, as 40 (17.4%) participants continued to assess themselves as at least very mildly sick at the end of the maximum 14 day monitoring period.

Colds tend to begin with specific nasal or throat symptoms, or with nonspecific or general feelings of tiredness or malaise, sometimes difficult to quantify in terms of onset timing. In this sample, 97 (42%) people reported a sore or scratchy throat as their first symptom, with 105 (46%) reporting nasal discharge, obstruction or sneezing, and only 7 (3%) reporting cough as their first symptom. At enrollment, less than 48 hours from first symptom, 223 (97%) reported at least one nasal symptom, 201 (87%) had sore throat, and some 150 (65%) reported cough. Nonspecific symptoms were also highly prevalent, with 142 (62%) reporting headache, 87 (38%) chilliness, and 184 (80%) malaise, tiredness or lack of energy.

Severity of illness at enrollment varied greatly across all measures: WURSS-44, Jackson, and SF-8. Means, (standard deviations), and [interquartile ranges] were as follows: 9.54, (3.68), [7,12] for Jackson, 100.6, (51.2), [59, 134] for the WURSS-44, 40.3 (9.42) [33.3, 47.7] for SF-8 physical health, and 47.1 (9.34) [42.4, 54.4] for SF-8 mental health. Corresponding values for the WURSS global-severity-today item at enrollment were 4.10, (1.26), [3,5] Summary scores for the WURSS-44 and WURSS-21 are simple sums of all responses except the introductory global-severity-today score and the concluding global-change-since-yesterday items. This deviates from first reporting of WURSS validity,[24] where global-severity-today was included in the summary score. We have since decided that "How sick do you feel today?" and "Please rate the average severity of your cold symptoms over the last 24 hours" refer to conceptually distinct time frames and hence should be not be lumped together in summary scores.

The pattern of experienced symptoms was characterized by the expected high frequency reporting of nasal symptoms (99.6%), sore or scratchy throat (97.8%), and cough (93.5%), reported at least once during the first seven days of illness. Sinus symptoms were also widely reported (92.2%), as were headache (89.6%) and body aches (88.7%). Other frequently reported symptoms were referable to the chest (73.9%), ears (77.0%), and eyes (83.5%). Swollen glands (67.4%), chilliness (63.9%) and feverishness (73.0%) were also experienced frequently. All N = 230 (100%) of our participants scored themselves as having some degree of tiredness, malaise, or feeling run down at least once during up to 7 days of illness. Some degree of functional limitation was also reported by 100% of our sample, with the following abilities receiving impairment scores above zero at least once during the first seven days of illness: think clearly (90%), speak clearly (83.5%), sleep well (91.3%), breathe easily (95.7%), accomplish daily activities (90.0%), interact with others (87.8%), and live your personal life (88.7%). The WURSS uses "very mild" as a response option. Frequency of items rated as mild, moderate or severe were somewhat lower.

**Figure 1:**
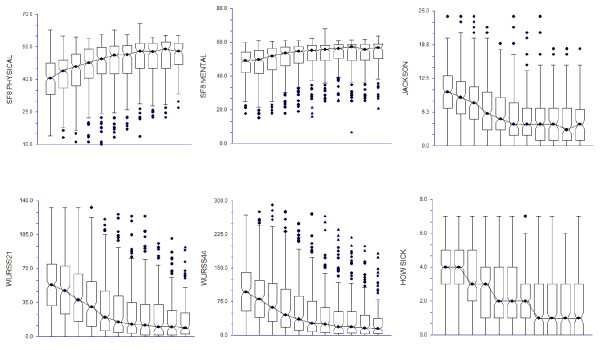
**Data shown represent Day 2 to Day 12**. Sample size diminishes as participants’ colds resolve, from N=228 on Day 2 to N=100 on Day 12. The center of the notched boxes is the median summed score for that day.  The notches portray the median ± 1.57 (interquartile range=IQR) / N^-2^ and thus can be compared to assess difference at the P = 0.05 level of significance. The top of the notched boxes indicate the 25% and 75% percentiles, respectively.  The ends of the vertical lines indicate the last actual data point within 1.5 (IQR) from the 25%ile and 75%ile. The symbols above and below these lines are actual outlying data points.

Figure 1 shows daily change over time of illness severity as measured by the WURSS-21, the WURSS-44, the Jackson scale, and the SF-8 (both physical and mental health scores). Sample size decreases as participants report resolution of their illnesses, from N = 230 on Day 1 to N = 100 on Day 12, as only those with continuing colds are included. Day-to-day change would appear even more dramatic if those reporting resolution of illness were included in these figures. As measured by the SF-8, general physical health is impaired more and recovers more swiftly than mental health during common cold illness. Illness-specific health changes more rapidly than general health, whether measured by Jackson symptoms or by either version of WURSS. All changes are more rapid in the first several days than later on.

**Figure 2:**
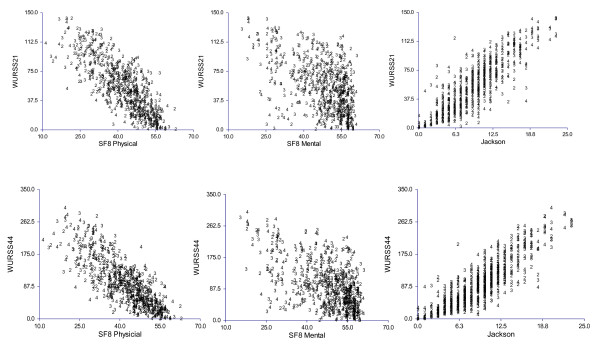
**Data shown represent Days 2, 3 and 4, where sample size was N = 228, N = 226 and N = 224, respectively**. Day 3 Pearson correlations (95% confidence intervals) against the WURSS-21 were 0.925 (0.903, 0.942) for the WURSS-44, 0.849 (0.808, 0.882) for Jackson, -0.793 (-0.739, -0.837) for SF-8 physical, and -0.547 (-0.448, -0.632) for SF-8 mental. Correlations to the WURSS-44 were 0.879 (0.846, 0.906) for Jackson, -0.799 (-0.746, -0.842) for SF-8 physical, and -0.599 (-0.507, -0.677) for SF-8 mental. Jackson correlated to SF-8 physical at -0.748 (-0.684, -0.800) and to SF-8 mental at -0.555 (-0.457, -0.640). All associations were statistically significant at p < 0.001.

Figure 2 shows scatterplot correlations of the WURSS-21 and WURSS-44 with SF-8-assessed general physical and mental health, and with the Jackson score. Illness-specific health-related quality-of-life (WURSS) correlates more closely with physical than mental health, as expected. Jackson symptoms also correlate more strongly with SF-8 physical than mental health. Both versions of WURSS associate more strongly with Jackson and SF-8 than those two measures do with each other. Not unexpectedly, the strongest associations observed were the WURSS-21 with its parent WURSS-44, yielding Pearson correlation coefficients of 0.920, 0.925, and 0.937 on Days 2, 3 and 4, respectively. Together, we interpret these findings as evidence of convergent validity.

Tables 3 and 4 present item-by-item evaluation criteria for the WURSS-44 and WURSS-21. Each item is portrayed in terms of frequency, severity, minimal important difference (MID), mean squared error (MSE), used to generate Guyatt's responsiveness coefficient. Coefficients representing these criteria are strikingly similar to those in the first WURSS validation study[24]. WURSS-21 items also appear to perform similarly when included in the WURSS-44, and when rated separately in the short form WURSS-21. In general, items included in the WURSS-21 demonstrate greater responsiveness than the WURSS-44 items not included in the 21-item version. One exception is that WURSS-44 items #13 (feeling "run down") and #32 (lack of energy) perform very well, but are not included in the WURSS-21. When similar findings were noted in the first validation study, we decided not to include these in the short form WURSS-21 because of excessive overlap (redundancy) with item #18 (feeling tired). The instruments as a whole yielded similar MIDs and responsiveness indices to the first study,[24] with MID and responsiveness index of 18.5 and 0.75 for the WURSS-44, and 10.3 and 0.71 for the WURSS-21 in the current study, compared to 16.7 and 0.71 for the WURSS-44 and 9.48 and 0.80 for the WURSS-21 (as 19 items embedded in the WURSS-44) in the first study[24].

**Table 3:** Frequency, severity, importance, minimal important difference and responsiveness of WURSS-44 Items

**Item**	**Frequency**	**Severity**	**Importance**	**MID**	**MSE**	**Responsiveness**
***1***	***100.0***	***4.14 ± 1.42***	***4.06 ± 0.88***	***0.73***	***0.89***	***0.55***

***2***	***90.0***	***3.71 ± 1.77***	***3.31 ± 1.15***	***0.43***	***1.08***	***0.29***

3	81.3	3.74 ± 1.84	3.45 ± 1.28	0.36	1.39	0.21

4	73.5	3.93 ± 1.92	4.26 ± 1.16	0.39	1.51	0.23

***5***	***93.0***	***3.77 ± 1.82***	***3.67 ± 1.09***	***0.50***	***1.20***	***0.32***

***6***	***95.2***	***3.82 ± 1.79***	***3.36 ± 1.15***	***0.49***	***1.51***	***0.28***

***7***	***87.8***	***3.56 ± 1.83***	***3.09 ± 1.30***	***0.46***	***1.14***	***0.30***

***8***	***98.3***	***4.04 ± 1.63***	***3.71 ± 1.13***	***0.53***	***1.56***	***0.30***

***9***	***96.5***	***4.04 ± 1.77***	***3.76 ± 1.18***	***0.49***	***1.61***	***0.27***

***10***	***96.1***	***3.22 ± 1.73***	***3.04 ± 1.24***	***0.52***	***1.71***	***0.28***

11	89.6	3.93 ± 1.70	4.14 ± 1.05	0.47	1.50	0.27

12	88.7	3.66 ± 1.84	3.89 ± 1.10	0.48	1.45	0.28

13	99.1	4.36 ± 1.74	4.39 ± 0.87	0.73	1.48	0.42

14	60.9	3.61 ± 1.91	2.98 ± 1.25	0.26	0.95	0.19

15	63.9	3.37 ± 1.78	3.13 ± 1.26	0.29	1.15	0.19

16	73.0	3.64 ± 1.82	3.54 ± 1.22	0.37	1.55	0.21

17	70.0	3.20 ± 1.83	3.69 ± 1.25	0.25	1.54	0.14

***18***	***100.0***	***4.21 ± 1.84***	***4.10 ± 01.03***	***0.70***	***1.33***	***0.43***

19	89.6	3.42 ± 1.81	3.55 ± 1.04	0.35	1.54	0.20

20	77.0	3.48 ± 1.72	3.47 ± 1.29	0.42	1.71	0.23

21	84.8	3.59 ± 1.68	3.38 ± 1.25	0.48	1.52	0.27

22	90.9	3.73 ± 1.66	3.41 ± 1.17	0.53	1.55	0.30

23	67.4	3.47 ± 1.73	2.97 ± 1.32	0.28	0.85	0.21

24	72.6	3.45 ± 1.67	3.19 ± 1.32	0.37	0.98	0.27

25	70.4	3.35 ± 1.76	3.39 ± 1.27	0.30	1.56	0.17

26	77.0	3.30 ± 1.89	2.77 ± 1.28	0.32	0.79	0.25

27	73.5	3.32 ± 1.77	3.16 ± 1.26	0.29	1.10	0.20

***28***	***93.5***	***3.99 ± 1.62***	***3.75 ± 1.04***	***0.55***	***1.96***	***0.28***

***29***	***70.9***	***3.56 ± 1.88***	***3.49 ± 1.20***	***0.37***	***0.97***	***0.26***

30	60.0	3.46 ± 1.72	3.32 ± 1.25	0.28	0.75	0.23

31	60.4	3.43 ± 1.78	3.21 ± 1.29	0.25	0.89	0.19

32	98.7	4.30 ± 1.86	4.31 ± 0.90	0.68	1.38	0.41

33	83.9	3.49 ± 1.79	2.84 ± 1.41	0.39	1.08	0.27

***34***	***90.0***	***3.45 ± 1.71***	***4.47 ± 0.87***	***0.51***	***0.75***	***0.42***

35	83.5	3.35 ± 1.77	4.00 ± 1.15	0.39	1.33	0.24

***36***	***91.3***	***4.23 ± 1.82***	***4.59 ± 0.82***	***0.55***	***1.45***	***0.32***

***37***	***95.7***	***3.90 ± 1.81***	***4.35 ± 0.94***	***0.59***	***1.21***	***0.38***

***38***	***87.0***	***3.72 ± 1.84***	***3.93 ± 1.11***	***0.49***	***1.25***	***0.31***

***39***	***90.0***	***3.55 ± 1.74***	***4.26 ± 0.98***	***0.55***	***0.98***	***0.39***

***40***	***81.7***	***3.87 ± 1.82***	***3.93 ± 1.27***	***0.46***	***0.99***	***0.33***

***41***	***85.2***	***3.54 ± 1.80***	***3.75 ± 1.14***	***0.46***	***0.93***	***0.34***

***42***	***87.8***	***3.29 ± 1.75***	***4.08 ± 1.02***	***0.44***	***1.18***	***0.28***

***43***	***88.7***	***3.49 ± 1.83***	***4.28 ± 1.01***	***0.53***	***1.12***	***0.35***

Items selected for the ***WURSS-21 ***are displayed in ***bold italics***

The first and last items on both the WURSS-21 and WURSS-44 differ from other items in terms of purpose and recall period, hence are not included in summary scores.

Frequency = Scored above zero at least once in first seven days of monitoring,

Severity = Mean severity on 7-point scale averaged over first three days; Calculated only for those with symptom present all three days. To weight each person's responses equally, data were first averaged within-person-over-time, then averaged among participants

Importance = Items were rated for importance on a 5-point scale at intake only, and only on the WURSS-44

MID = Minimal Important Difference = Mean day-to-day change for those rating themselves as "a little better" or "somewhat better" compared to the last time they filled out the questionnaire

MID and Guyatt's responsiveness index were 10.3, 0.71 for the WURSS-21 and 18.5, 0.75 for the WURSS-44, respectively

MSE = Mean squared error for all people who rated themselves as "the same" for two days in a row

**Table 4:** Frequency, severity, minimal important difference, and responsiveness of WURSS-21 Items

**Symptom**	**Item # on W-21**	**Item# on W-44**	**Frequency**	**Severity**	**MID**	**MSE**	**Responsiveness**
How sick	1	1	100.0	4.13 ± 1.46	0.77	0.78	0.62

Runny nose	2	8	98.3	3.70 ± 1.77	0.56	1.48	0.33

Plugged nose	3	9	96.5	4.00 ± 1.79	0.57	1.54	0.32

Sneezing	4	10	95.7	3.34 ± 1.76	0.50	1.20	0.32

Sore throat	5	5	92.6	3.76 ± 1.85	0.49	1.00	0.35

Scratchy throat	6	6	96.1	3.82 ± 1.81	0.50	1.25	0.32

Cough	7	2	92.2	3.80 ± 1.84	0.46	1.76	0.25

Hoarseness	8	7	86.1	3.38 ± 2.01	0.41	1.29	0.26

Head congestion	9	28	93.0	4.03 ± 1.70	0.64	1.54	0.37

Chest congestion	10	29	75.7	3.76 ± 1.88	0.38	0.97	0.27

Feeling tired	11	18	99.6	4.33 ± 1.80	0.82	1.41	0.49

Think clearly	12	34	91.3	3.53 ± 1.68	0.54	1.02	0.38

Sleep well	13	36	93.9	4.17 ± 1.82	0.66	1.69	0.36

Breathe easily	14	37	96.5	3.84 ± 1.86	0.60	1.08	0.41

Walk/Climb stairs	15	38	89.6	3.75 ± 1.81	0.50	0.88	0.38

Accomplish daily activities	16	39	90.4	3.57 ± 1.74	0.57	1.08	0.39

Work outside the home	17	40	82.2	3.80 ± 1.84	0.48	1.16	0.32

Work inside the home	18	41	87.0	3.52 ± 1.81	0.51	0.80	0.40

Interact with others	19	42	86.5	3.50 ± 1.73	0.53	0.93	0.39

Live your personal life	20	43	88.3	3.58 ± 1.74	0.58	0.92	0.43

The first and last items on both the WURSS-21 and WURSS-44 differ from other items in terms of purpose and recall period, hence are not included in summary scores.

Frequency = Scored above zero at least once in first seven days of monitoring,

Severity = Mean severity on 7-point scale averaged over first three days; Calculated only for those with symptom present all three days. To weight each person's responses equally, data were first averaged within-person-over-time, then averaged among participants

Importance = Items were rated for importance on a 5-point scale at intake only, and only on the WURSS-44

MID = Minimal Important Difference = Mean day-to-day change for those rating themselves as "a little better" or "somewhat better" compared to the last time they filled out the questionnaire

MID and Guyatt's responsiveness index were 10.3, 0.71 for the WURSS-21 and 18.5, 0.75 for the WURSS-44, respectively

MSE = Mean squared error for all people who rated themselves as "the same" for two days in a row

Arguably, importance-to-patients may be the most valuable criteria for determining which items should be included in any health-assessing questionnaire. Analysis of responses regarding importance confirmed and extended the findings from our previous WURSS validity study. Mean importance of items ranged from 2.77 (watery eyes) to 4.59 (sleep well) on a 1 to 5 scale, with very similar patterns to those found in the first study. Another previously noted finding is that functional quality-of-life items tend to be rated as more important than items rating symptoms. Among symptom-assessing items, the more frequent (nasal, sore throat, cough, head congestion, chest congestion) tend to be rated as more important than those less frequent (sweats, chills, swollen glands, eye symptoms). Overall, the majority of WURSS items, especially those selected for the WURSS-21, were rated as at least "somewhat important" by most of the people most of the time.

Tables 5, 6 and 7 show the results of factor analysis for the WURSS-44, and tables 8, 9 and 10 display corresponding results for the WURSS-21. Exploratory analysis began with Day 3 data, chosen because this day represents the breadth of symptomatic and functional impairment as well or better than any other day. Factorial structures were fit allowing for three to 43 dimensions for the WURSS-44. Very little added explanatory power was found for models with nine or more dimensions, hence we settled on an eight dimension model. For the WURSS-21, a 3-dimensional structure was chosen, after looking at fit indices for models with two to 20 dimensions. Tables 6 and 9 show additional coefficients for the models selected, as well as indicators of how these factorial models play out over time. Fit indices for both instruments are strong, easily meeting criteria suggested by Hu and Bentler[58]. Tables 7 and 10 show individual items in the dimensional structures, along with indicators of reliability. Reliability coefficients derived by methods of Joreskog[51] and Bollen[52] were all significant at p < 0.01 using Wald testing[55,56].

**Table 5:** Model fit Exploratory Factor Analysis for WURSS-44 using 3 to 10 dimensions

Dimensions	Chi-square	df	χ^2^/df	CFI	TLI	RMSEA	SRMR
3	6902.7	738	9.35	.974	.970	.192	.076

4	5114.1	699	7.31	.982	.977	.167	.061

5	3946.8	661	5.97	.986	.982	.148	.050

6	2922.0	624	4.68	.990	.987	.127	.041

7r	2054.4	489	4.20	.993	.989	.119	.034

8	1785.5	553	3.22	.995	.992	.099	.029

8r	1625.9	457	3.55	.995	.992	.106	.028

9	1430.5	519	2.75	.996	.994	.088	.024

10	1165.6	486	2.39	.997	.995	.078	.021

7r and 8r = Dimensions restricted to exclude headache, loss of appetite, and sleep well,

items which did not

CFI = Comparative Fit Index

TLI = Tuker-Lewis Index

RMSEA = Root Mean Square Error of Approximation

SRMR = Standardized Root Mean Square Residual

Hu and Bentler (1999)[58] suggest the following cut off values for good fit, CFI > .95, TLI > .95, RMSEA < .06, and SRMR < .08

**Table 6:** Best fit factorial model for WURSS-44

CFA Final model structure for the WURSS-44 at day 3				
Dimensions 8 restricted	Chi-square	df	χ^2^/df	CFI
	
	296.2	69	4.29	.951

Number of items used = 34		TLI	RMSEA	WRMR

		.991	.120	.975

Time invariance (configural invariance) Days 2 to 7				

Day	CFI	TLI	RMSEA	WRMR

2	.935	.985	.123	1.040

3*	.951	.991	.120	.975

4	.957	.992	.119	.913

5	.972	.995	.105	.843

6	.982	.995	.106	.856

7	.974	.995	.097	.761

3* = Data from day 3 was used for original model

CFA = Confirmatory Factor Analysis

CFI = Comparative Fit Index

TLI = Tuker-Lewis Index

RMSEA = Root Mean Square Error of Approximation

WRMR = Weighted Root Mean Square Residual

Hu and Bentler (1999)[58] suggest the following cut off values for good fit, CFI > .95, TLI > .95, RMSEA < .06, and WRMR < .90

**Table 7:** Best fit factorial model for WURSS-44

**Throat issues with cough**		**Sinus**	
Composite reliability	0.895	Composite reliability	0.939

AVE	0.61	AVE	0.770

Cronbach's α	0.907	Cronbach's α	0.897

Items in dimension	Loading	Items in dimension	Loading

Coughing	0.243	Sinus pain	0.923

Coughing stuff up	0.224	Sinus pressure	0.952

Cough interfering with sleep	0.337	Sinus drainage	0.883

Sore throat	0.952	Head congestion	0.580

Scratchy throat	0.890	Chest congestion	0.255

Hoarseness	0.854		
		
Speak clearly	0.449		

**Sweats**		**Ear and Eye Issues**	

Composite Reliability	0.927	Composite reliability	0.901

AVE	0.760	AVE	0.740

Cronbach's α	0.871	Cronbach's α	0.852

Items in dimension	Loading	Items in dimension	Loading

Sweats	0.885	Plugged ears	0.941

Chills	0.895	Ear discomfort	0.943

Feverish	0.880	Watery eyes	0.295

Dizziness	0.828	Eye discomfort	0.274

**Tiredness**		**Cough with Chest Issues**	

Composite reliability	0.959	Composite reliability	0.956

AVE	0.867	AVE	0.780

Cronbach's α	0.939	Cronbach's α	0.929

Items in dimension	Loading	Items in dimension	Loading

Feeling run down	0.977	Cough	0.712

Feeling tired	0.959	Coughing stuff up	0.676

Lack of energy	0.970	Cough interfering with sleep	0.636

Head congestion	0.354	Chest congestion	0.776

**Activity and function**		Chest tightness	

Composite reliability	0.967	Heaviness	0.957

AVE	0.790	**Nasal and Eye Issues**	

Cronbach's α	0.952	Composite reliability	0.873

Items in dimension	Loading	AVE	0.630

Think clearly	0.815	Cronbach's α	0.759

Walk, climb stairs, exercise	0.904	Items in dimension	Loading

Accomplish daily activities	0.973	Runny nose	0.760

Work outside the home	0.923	Sneezing	0.761

Work inside the home	0.970	Watery eyes	0.685

Interact with others	0.902	Eye discomfort	0.757

Live your personal life	0.922		
		
Speak clearly	0.393		

AVE = Average Variance Extracted

Loading = Standardized loading coefficient

**Table 8:** Model fit EFA for WURSS-21 using 2 to 7 dimensions

Dimensions	Chi-square	df	χ^2^/df	CFI	TLI	RMSEA	SRMR
2	1547.9	134	11.5	0.986	0.982	0.215	0.064

3	866.5	117	7.4	0.993	0.989	0.167	0.043

4	580.3	101	5.7	0.995	0.992	0.144	0.032

5	381.8	86	4.4	0.997	0.994	0.123	0.023

6	254.8	72	3.5	0.998	0.996	0.105	0.017

7	136.1	59	2.3	0.999	0.998	0.076	0.012

CFI = Comparative Fit Index

TLI = Tuker-Lewis Index

RMSEA = Root Mean Square Error of Approximation

SRMR = Standardized Root Mean Square Residual

Hu and Bentler (1999)[58] suggest the following cut off values for good fit, CFI > .95, TLI > .95, RMSEA < .06, and SRMR < .08

**Table 9:** Best fit factorial model for WURSS-21

CFA Final model structure of the WURSS-21 at day 3				
Dimensions 3 restricted	Chi-square	df	χ^2^/df	CFI
	
	245.7	37	6.6	0.949

Number of items used = 20		TLI	RMSEA	WRMR

		0.990	0.157	1.074

Time invariance (configural invariance) Days 2 to 7				

Day	CFI	TLI	RMSEA	WRMR

2	0.903	0.978	0.170	1.234

3*	0.949	0.990	0.157	1.074

4	0.962	0.993	0.157	1.047

5	0.970	0.995	0.145	0.973

6	0.983	0.995	0.147	1.030

7	0.980	0.995	0.132	0.909

3* = D ata from day 3 was used for original model

CFI = Comparative Fit Index

TLI = Tuker-Lewis Index

RMSEA = Root Mean Square Error of Approximation

WRMR = Weighted Root Mean Square Residual

Hu and Bentler (1999)[58] suggest the following cut off values for good fit, CFI > .95, TLI > .95, RMSEA < .06, and WRMR < .90

**Table 10:** Best fit factorial model for WURSS-21

**Nasal**	
Composite Reliability	0.922

AVE	0.578

Cronbach's α	0.912

Items in dimension	Loading

Runny nose	0.618

Plugged nose	0.744

Sneezing	0.648

Cough	0.521

Head congestion	0.837

Chest congestion	0.854

Feel tired	0.467

Sleep well	0.848

Breathe easily	0.874

**Throat**	

Composite Reliability	0.903

AVE	0.725

Cronbach's α	0.881

Items in dimension	Loading

Sore Throat	0.948

Scratchy Throat	0.903

Cough	0.285

Hoarseness	0.875

**Activity and function**	

Composite Reliability	0.972

AVE	0.821

Cronbach's α	0.961

Items in dimension	Loading

Feel tired	0.422

Think clearly	0.820

Walk, climb stairs, exercise	0.903

Accomplish daily activities	0.975

Work outside of home	0.912

Work inside of home	0.969

Interact with others	0.927

Live your personal life	0.939

AVE = Average Variance Extracted

Loading = Standardized loading coefficient

Table 11 displays estimated sample size for two-armed randomized trials, using data gathered here, and common statistical assumptions used in power studies. Powering a common cold treatment trial on MID and responsiveness makes most sense when the therapy is hypothesized to influence the rate of recovery, and when trialists prefer to study participants for a week or less. The main limitation is that MID and daily change rates are neither intuitive nor supported by theory as primary outcomes. Powering a trial on area-under-the-curve makes more sense from a theoretical perspective, as overall illness-related quality-of-life is an intuitively understandable and conceptually consistent primary outcome. For the sample described here, mean AUC for the WURSS-21 was 310.1 with standard deviation 251.0. Corresponding values for the WURSS-44 were mean 570.6 and SD 504.5.

**Table 11:** Sample size for powering trials using WURSS-21 and WURSS-44

	one-tailed α = 0.005 (two-tailed α = 0.01)			one-tailed α = 0.025 (2-tailed α = 0.05)			one-tailed α = 0.05 (two-tailed α = 0.10)		
β =	0.05	0.10	0.20	0.05	0.10	0.20	0.05	0.10	0.20

Power	95%	90%	80%	95%	90%	80%	95%	90%	80%

Sample size per group needed to detect day-to-day MID (using Guyatt's responsiveness coefficient)									

WURSS-21	72	60	47	52	43	32	44	35	25

WURSS-44	64	53	42	47	38	28	39	31	22

WURSS-21 – Sample size per group needed to detect between group AUC differences of:									

10%	2348	1961	1540	1712	1385	1035	1426	1129	815

20%	578	483	379	421	341	255	351	278	201

30%	259	217	171	189	153	115	157	124	90

40%	147	123	97	107	87	65	89	71	51

50%	95	80	63	69	56	42	57	46	33

WURSS-44 – Sample size per group needed to detect between group AUC differences of:									

10%	2787	2328	1828	2033	1644	1228	1693	1340	967

20%	697	583	458	508	411	307	423	335	242

30%	312	261	205	227	184	138	189	150	108

40%	176	147	116	128	104	78	107	85	61

50%	113	95	75	82	67	50	69	55	40

## Discussion

The current study confirms that the Wisconsin Upper Respiratory Symptom Survey, in both 44-item and 21-item format, demonstrates broad-based construct validity. Original item selection came from open-ended questions eliciting terminology from people with self-identified colds[23]. When three or more people identified a specific symptomatic or functional impact, an item was included in theWURSS-44. That instrument was then tested among 150 adults during 1,681 person-days of common cold illness, and demonstrated good reliability, responsiveness, and convergence with other measures[24]. Importance-to-patient and responsiveness were used as criteria to select a subset of items for a short form version, the WURSS-21. The current paper describes a third phase in WURSS validation, in which 230 people with colds were monitored for 2,457 person-days, filling out both the 44 and 21 item versions each day of illness. Results shown here demonstrate that the WURSS-44 performs similarly in different samples, and that the WURSS-21 demonstrates approximately the same performance criteria as the parent WURSS-44.

Overall, the results are encouraging. Coefficients representing reliability, responsiveness, and importance-to-patients are similar to those from the previous study. Items selected for the WURSS-21 perform similarly whether embedded within the WURSS-44 or separately in the WURSS-21. Convergence with external comparators (SF-8, Jackson) follows predictions from theory and previous experience. Our qualitative experience talking with research participants tells us that one reason the WURSS performs well is that it was designed to be user-friendly, with easy-to-understand questions and response ranges. Consideration of face validity tells us that WURSS is a better measure than Jackson, as it includes items that rate functional impairment and quality-of-life, which have been rated as important by people suffering from colds.

Despite these strengths, there are of course limitations. The original item-generation procedures may have failed to include representation of cold-related symptoms or functional impairments that are important to significant proportions of cold-sufferers. Alternative wording, formatting, and response range options have not been developed or tested. All of the work has been done in and around Madison Wisconsin, which may influence both the types of colds studied, and the linguistic and health value orientations of the population sampled. Finally, and perhaps most importantly, there are no gold standards for identifying, classifying, or assessing acute viral respiratory infections, hence criterion validity is not possible, and concepts such as sensitivity, specificity, and positive and negative predictive value cannot be used with confidence.

Following Guyatt, [25-29] we accept that the concepts of important difference and responsiveness are critical for assessing evaluative instruments, and have previously discussed related theory and methods in an article entitled: "Comparison of anchor-based and distributional approaches in estimating important difference in common cold"[45]. That paper compared MID to standardized effect size (ES) and standard error of measurement (SEM) as options to consider when seeking to evaluate change over time. Responsiveness, however, is not entirely satisfying for assessment of acute illness, which by definition has a beginning and an end, and thus both up sloping and down sloping severity curves. Deciding which time points to compare is not an easy task, as any specific choice brings with it corresponding limitations. To avoid severity-over-time complexities, some investigators may wish to use area under the severity duration curve (AUC) as the primary outcome for between-group comparison[59]. For these reasons, we have provided AUC descriptive statistics for the current study.

While it is clear that both versions of WURSS demonstrate broad-based construct validity, less confidence exists regarding underlying dimensional structure. The current study suggests an 8-dimensional structure for the WURSS-44, somewhat different from the 10-dimensional structure found in the first study. Factor analysis of the WURSS-21 in the current study suggests a 3-dimensional structure, substantially different from either of the two structures found for the WURSS-44. Perhaps this should not be too surprising, as dimensional representation was not used as criteria for deriving the short form. Nevertheless, we conclude that we have not yet reached confirmation of the true dimensional structure of either instrument, and thus cannot yet make recommendations regarding potential weighting of items within dimensions. Thus, we continue to recommend a simple sum of 42 items for the WURSS-44, and 19 items for the WURSS -21, as the most appropriate global severity score for these instruments. The first and last items are conceptually distinct, and hence should be analyzed and reported separately.

In conclusion, the data presented here confirms the construct validity of the WURSS-44, and extends these findings to the derivative short form, the WURSS-21. Both instruments remain free of charge for educational and non-profit use, and can be accessed through the website: 

## Competing interests

BB, RB and MM are authors and originators of the WURSS instrument, and hold partial copyrights administered by the Wisconsin Alumni Research Foundation (WARF). While WURSS is free for educational and nonprofit use, WARF may negotiate user fees for "for profit" use, with a portion returned to the author/originators. See .

## Authors' contributions

BB contributed to the design, supervised data collection and analysis, and wrote the manuscript.

RB contributed to the design, conducted statistical analysis, and contributed to the manuscript.

MM contributed to the design, conducted statistical analysis, and contributed to the manuscript.

GT coordinated data collection and contributed to the manuscript.

SB conducted data collection, and contributed to the manuscript.

AH entered, cleaned and analyzed data, and contributed to the manuscript.

MB entered and cleaned data, and contributed to the manuscript.

All authors have read and approved the final manuscript
